# The Silent Obstruction: A Rare Complication of Non-steroidal Anti-inflammatory Drug (NSAID)-Induced Diaphragm Disease

**DOI:** 10.7759/cureus.96275

**Published:** 2025-11-07

**Authors:** Edwin Mazhuppel Rechard, Ismaeel Al-Talib, Radhika Saigal, Lokesh Koumar Sivanandam

**Affiliations:** 1 Internal Medicine, The Royal Wolverhampton NHS Trust, Wolverhampton, GBR; 2 Gastroenterology, Walsall Healthcare NHS Trust, Walsall, GBR; 3 Internal Medicine, Walsall Manor Hospital, Walsall, GBR; 4 Internal Medicine, Torbay and South Devon NHS Foundation Trust, Torquay, GBR

**Keywords:** capsule retention, gastrointestinal stricture, small bowel diaphragm disease, small-bowel obstruction, small bowel resection

## Abstract

Diaphragm disease is a rare but significant complication of prolonged non-steroidal anti-inflammatory drug (NSAID) use, characterised by thin concentric mucosal diaphragms causing small bowel strictures.

This case report is on a 42-year-old woman with a chronic history of NSAID use, which led to the development of recurrent gastrointestinal symptoms, iron-deficiency anaemia, and subacute small bowel obstruction. Diaphragm disease was confirmed through histological analysis following surgical resection of the small bowel.

Patients who present with non-specific GI symptoms along with chronic NSAID use and unexplained obstruction should make us consider diaphragm disease within the differentials.

## Introduction

Diaphragm disease is an underrecognised complication of chronic non-steroidal anti-inflammatory drug (NSAID) use, characterised by the formation of multiple thin, concentric, diaphragm-like strictures made up of mucosa and submucosa with or without submucosal fibrosis in the small bowel, which eventually leads to a presentation classified as diaphragm-like strictures, which cause intermittent or complete small bowel obstruction [1-4]. 

It remains a rare diagnosis, often missed. Difficulty in diagnosis does arise due to its nonspecific clinical presentations (weight loss, abdominal pain, anaemia, diarrhoea, overt bleeding and perforation) and in visualising small bowel pathology with conventional modalities. It becomes clinically recognised when there is evidence of anaemia and hypoalbuminemia [1,2]. 

Small bowel diaphragm disease, first described in 1988 in the pathologic literature, is a relatively new clinical entity [2,3]. Long-term use of NSAIDs is considered the primary cause [4]. The pathogenesis of diaphragm disease is believed to result from NSAID-induced inhibition of prostaglandin synthesis, causing mucosal ischemia, ulceration, and subsequent submucosal fibrosis, ultimately producing diaphragm-like strictures [3]. Despite widespread NSAID use, the estimated prevalence among users is approximately 2% [1-3]. There are risk factors that contribute towards the formation of these strictures, which include female sex, prolonged use (as little as two months to several years), and advanced age [2-4]. 

We report a diagnostically challenging case of diaphragm disease, presenting with significant unintentional weight loss and non-specific gastrointestinal symptoms and malnutrition. This case has been reported in accordance with the SCARE 2025 criteria, ensuring a structured, comprehensive, and ethically sound report.

## Case presentation

**Figure 1:**
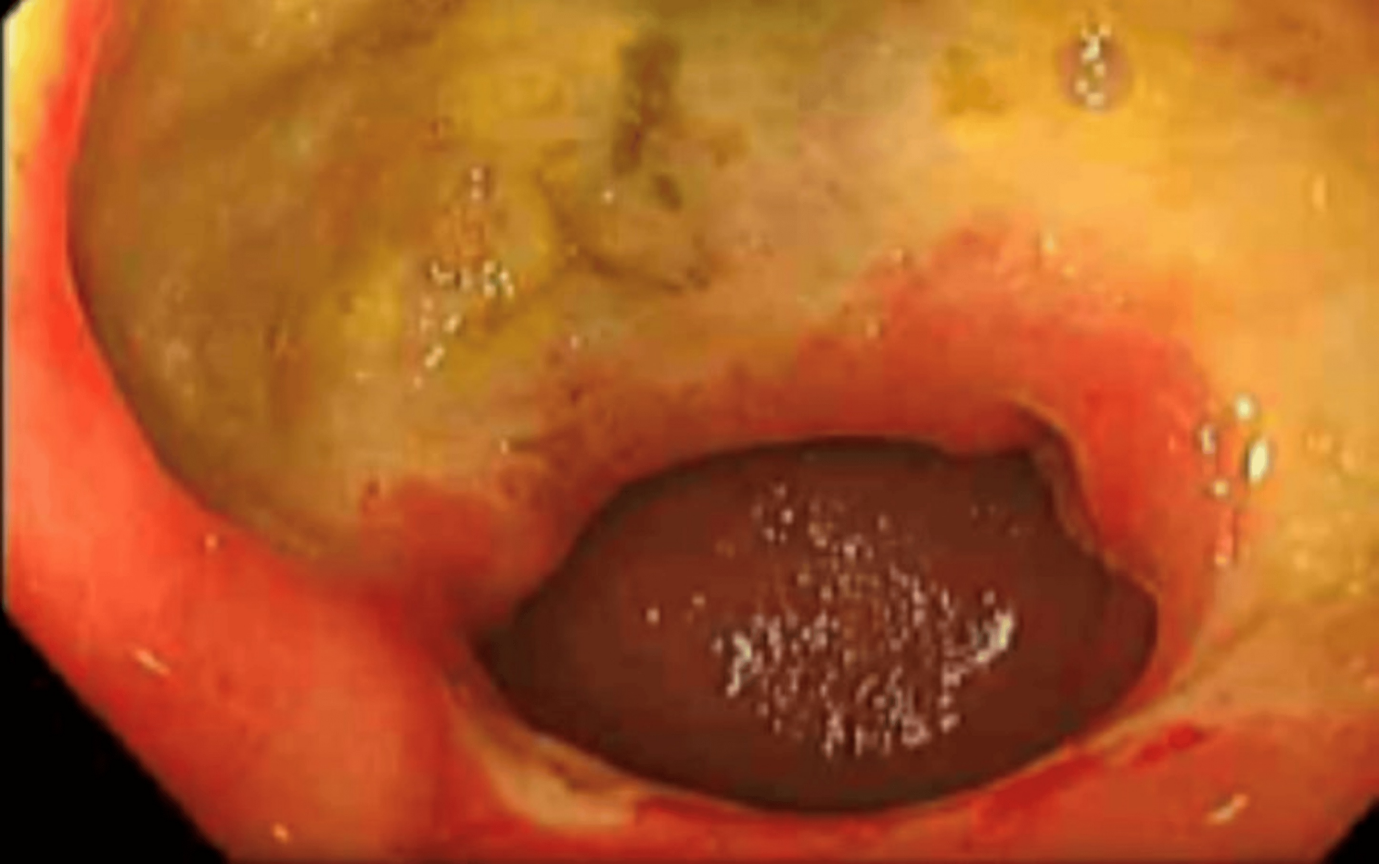
Large pyloric gastric ulcer

A 42-year-old woman presented to the emergency department with symptoms of abdominal pain, vomiting, weight loss and iron deficiency anaemia. She had no significant past medical history, but her medication history included ibuprofen and citalopram. She underwent an initial gastroscopy, which showed a large gastroduodenal (primarily pyloric) ulceration (Figure 1). The Campylobacter-like organism test was negative, and histology showed nonspecialised gastric-type mucosa with focal ulceration and no dysplasia. Given her significant weight loss and large ulcer, she underwent a CT scan of the thorax, abdomen and pelvis (CT TAP), which was unremarkable.

She had been taking ibuprofen 800mg/day for several months for symptomatic pain relief but had stopped for a few weeks prior to the presentation. She had no history of alcohol excess and no family history of gastrointestinal malignancy or inflammatory bowel disease. Her laboratory investigations on presentation revealed evidence of iron deficiency anaemia with hypoalbuminemia (albumin 26 g/L), normal IgG4 (0.11), and mildly elevated serum gastrin, which was likely due to proton pump inhibitor (PPI) use.

Despite treatment with high-dose PPIs, the ulcer persisted and worsened on subsequent gastroscopies (she had three repeat gastroscopies over eight months). 

A colonoscopy was arranged by the colorectal team due to a positive FIT test (faecal immunochemical test), which revealed chronic inflammation at the ileocecal (IC) valve with normal colonic and terminal ileum mucosa. A capsule endoscopy was performed six months after presentation to assess the small bowel. The small bowel capsule endoscopy was arranged with endoscopic deployment, which identified a deep, clean-based pyloric ulcer affecting about 50% of the pyloric channel circumference and a longitudinal ulcer involving approximately 20% of the circumference in the second part of the duodenum (D2). However, the small bowel preparation was poor with limited views of the distal small bowel, and the study was deemed incomplete. 

After the capsule endoscopy, she was reviewed in the clinic, where she reported a significant unintentional weight loss of over 10 kg in the past three months and a one-week history of nausea, vomiting, and abdominal pain. Clinical examination was unremarkable, with a soft, non-tender abdomen and no palpable masses. Abdominal X-ray showed no evidence of bowel obstruction, and the capsule was retained in the rectum.

Laboratory investigations of the clinic visit demonstrated anaemia, hypokalaemia and hypoalbuminemia (Table 1). She was admitted for nasojejunal feeding, intravenous iron infusion, and imaging (CT TAP).

**Table 1:** Clinic visit laboratory values

Parameter	Patient's Value	Reference Range
Haemoglobin	92 g/L	121 to 151 g/L
Potassium	2.8 mmol/L	3.5 to 5.5 mmol/L
Albumin	19 g/L	35 to 50 g/L
C-Reactive Peptide (CRP)	<1.0 mg/L	<1.0 mg/L
Iron	6.2 µmol/L	9 to 30 µmol/L

**Figure 2:**
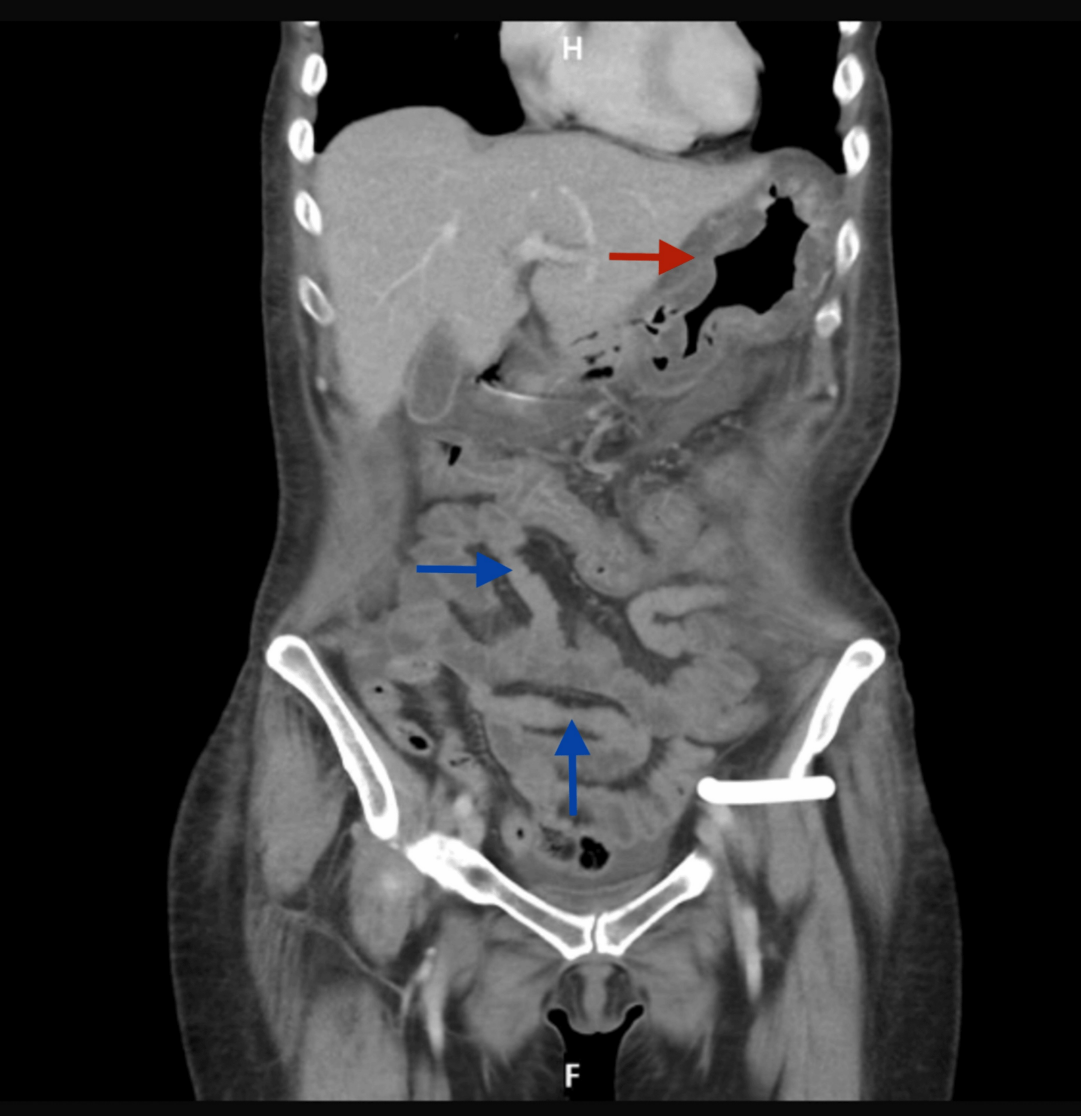
CT abdomen pelvis. The blue arrow points towards small bowel loops filled with fluids with intervening segment of collapsed bowel. The red arrow points towards diffuse gastric wall oedema, upper abdominal fat stranding, and mild ascites

Repeat CT thorax abdomen pelvis (Figure 2) revealed diffuse gastric wall oedema, upper abdominal fat stranding, and mild ascites without evidence of gastric outlet obstruction or hollow viscus perforation. These findings were consistent with anasarca. The case was discussed in the Upper Gastrointestinal multidisciplinary team meeting due to persistence of the ulcer. However, the current symptoms (obstructive symptoms with no evidence of gastric outlet obstruction on CT scan) with significant hypoalbuminemia raised the concern on small bowel pathology and recommended a repeat small bowel capsule endoscopy. 

**Figure 3:**
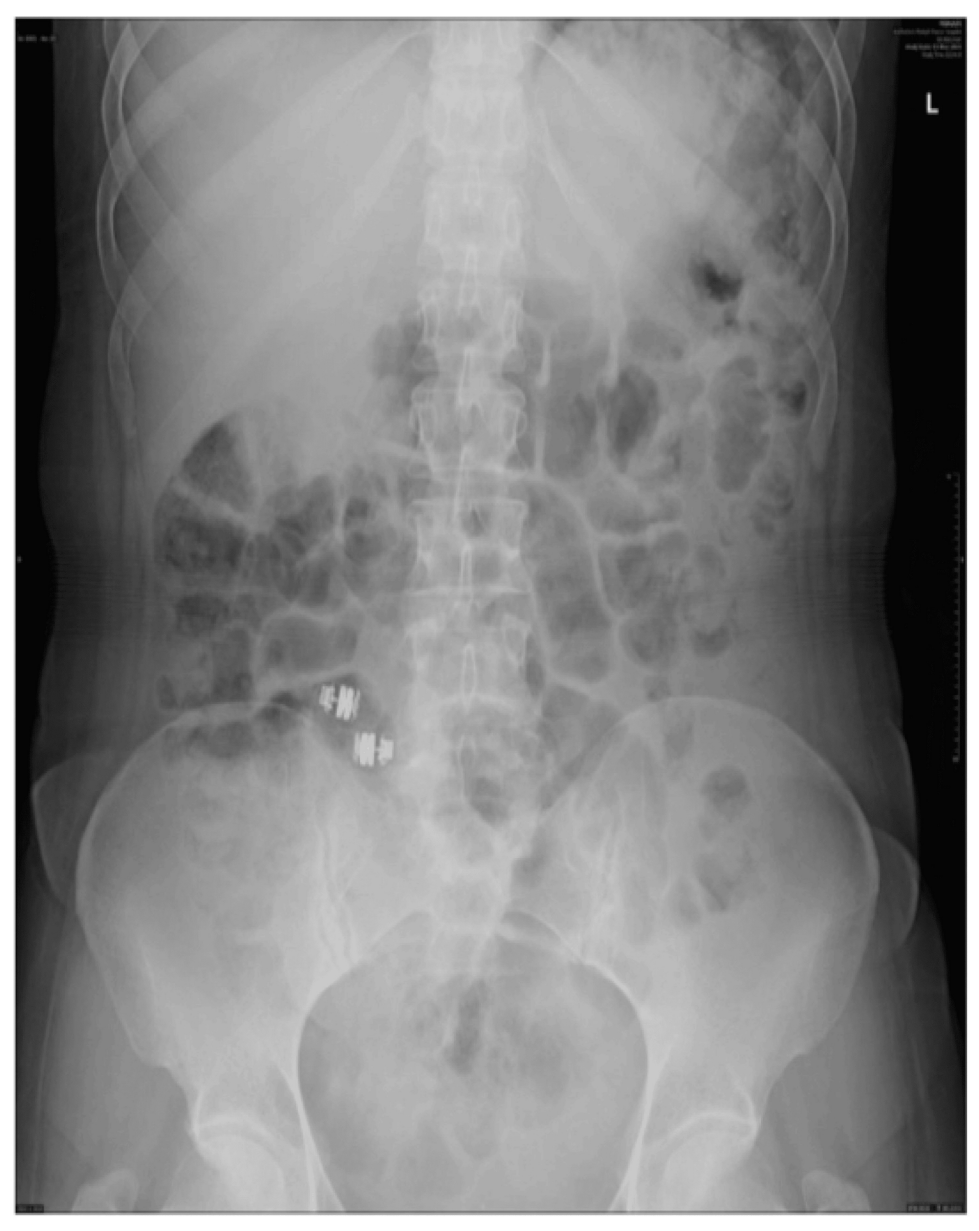
Abdominal X-ray showing two capsules retained at the terminal ileum

A repeat capsule endoscopy was arranged which showed a circumferential superficial ulcer/scar in the distal small bowel followed by a further stricture with more ulcerations. The capsule did not pass the second stricture, further raising the concern of capsule retention. Follow-up abdominal X-rays confirmed that both capsules were retained in the distal small bowel (Figure 3). 

Subsequent surgical review discussed options including surgical removal of retained capsules and the possibilities of small bowel resection or stricturoplasty. The patient underwent an elective small bowel resection with removal of both retained capsules. 

**Figure 4:**
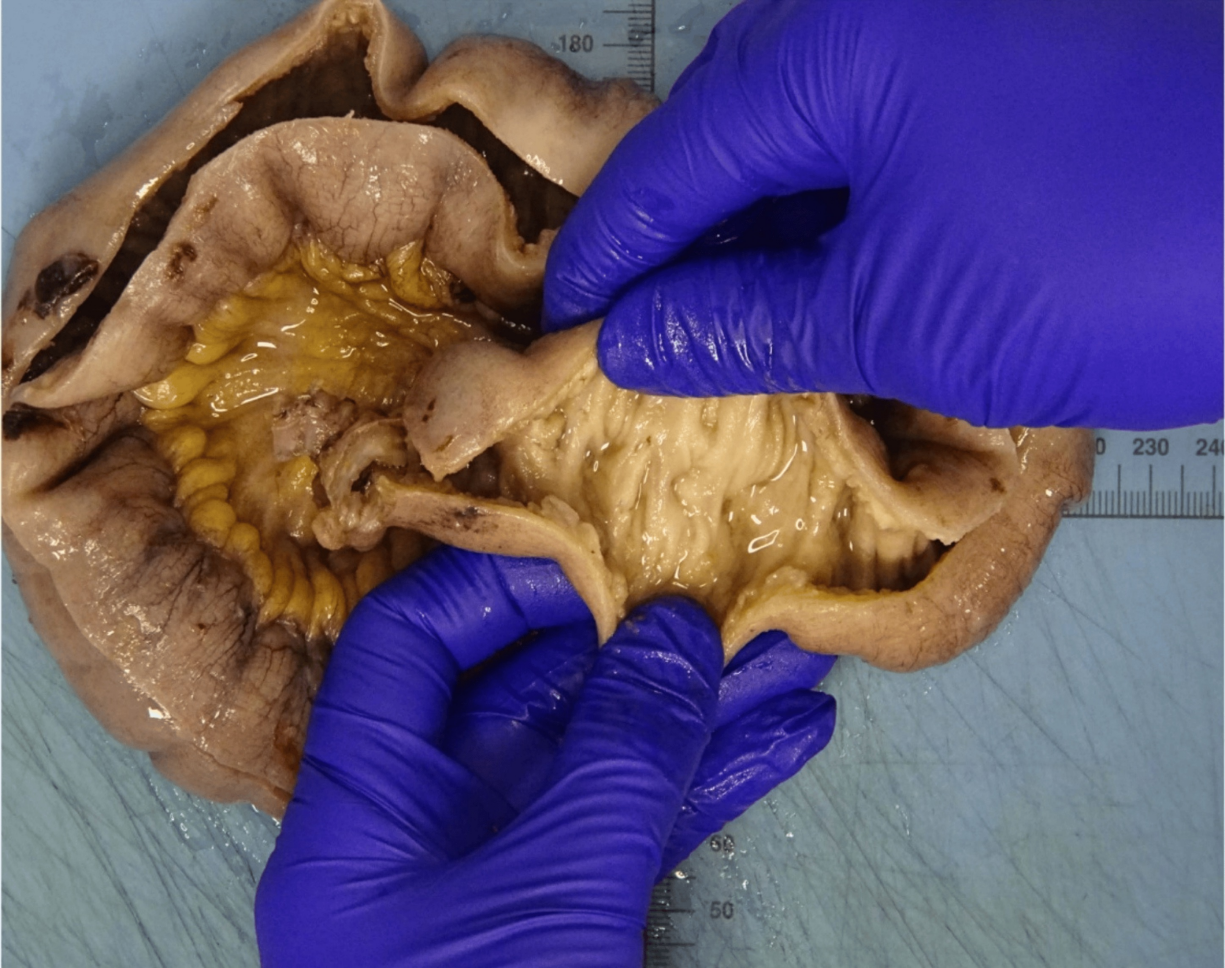
Macroscopic image showing segmentation of the small bowel with incomplete mucosal diaphragms

**Figure 5:**
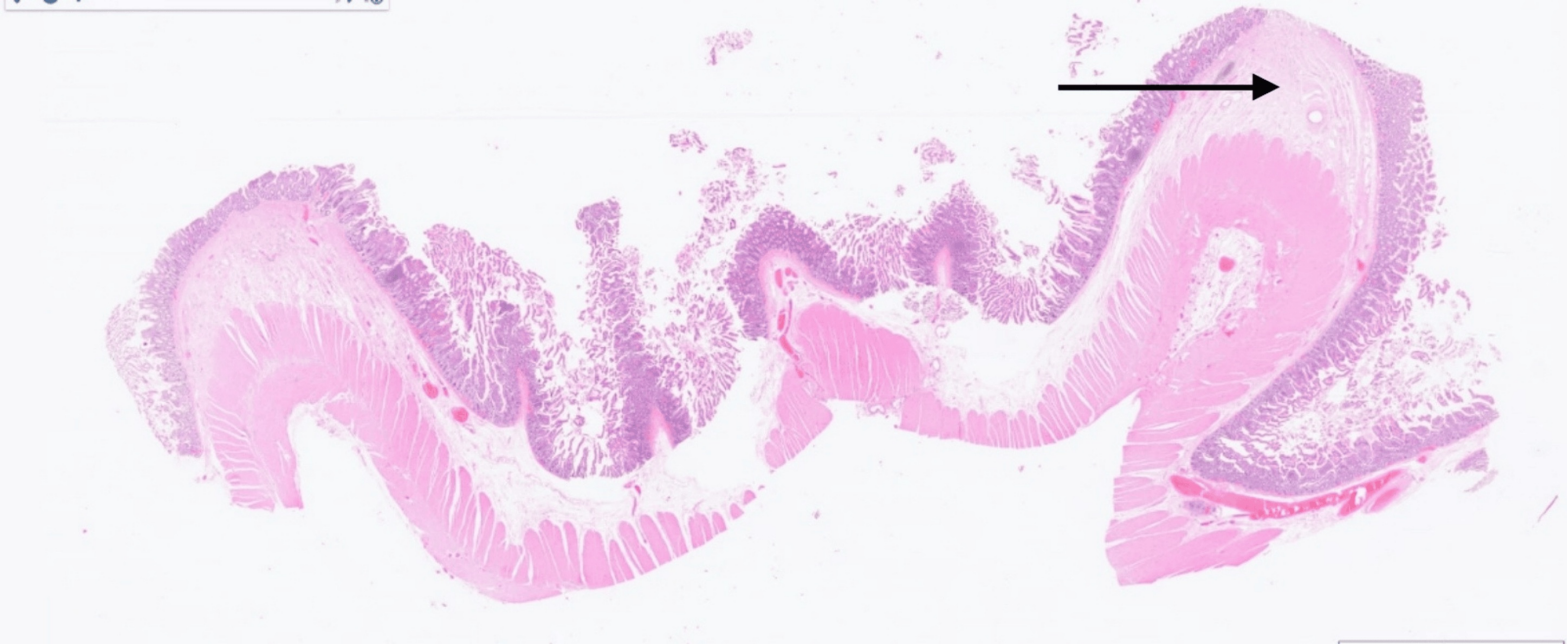
Microscopic image (H&E stain 0.5x) showing multiple incomplete thin circumferential mucosal diaphragms composed of fibrotic mucosa and submucosa. The black arrow pointing towards fibrosis

Histopathological analysis of the resected small bowel, both macroscopic (Figure 4) and microscopic (Figure 5), concluded sections of small bowel featuring segmentation by incomplete mucosal diaphragms, defined as thin circumferential membranes resembling the plica circularis, composed of mucosa and submucosa with accompanying fibrosis. Some of these show ulcerations of the tips. There is no evidence of transmural inflammation, fissures, granulomas, perforation, or malignancy. Mesenteric lymph nodes demonstrated no evidence of caseating granulomas and there was no evidence of Crohn's disease. 

These findings along with the clinical picture confirmed the diagnosis of NSAID-induced small bowel diaphragm disease. 

## Discussion

Diaphragm disease is a rare but serious complication of NSAID use, presenting variably with abdominal pain, anaemia, occult bleeding, non-specific gastrointestinal symptoms or obstruction. In our case, the finding of persistent gastro-duodenal ulcers did not explain the symptoms and clinical deterioration, with evidence of malabsorption suggesting a small bowel pathology. 

Diaphragm-like strictures have formed as a result of local inflammatory effects, which are linked to the formation of the reactive oxygen species due to the disruption of membrane phospholipids [4]. Diaphragm disease can affect both the small and large bowel and has been reported in 2% of patients taking long-term NSAIDs or COX-2 inhibitors [3]. In our case, there is a strong history of chronic NSAID use. 

In certain cases of diaphragm disease, CT scans have been reported to not show any specific abnormalities, as they lack the resolution required to highlight the diaphragm [4].

Although the use of video capsule endoscopy to aid the diagnosis of the condition has been reported, it runs a risk of retention of the capsule by 67%, especially when the lumen is significantly narrow [3,4]. Hence, it is not recommended if a patient presents with symptoms of obstruction. However, in our case, capsule endoscopy was performed because of the patient’s significant deterioration, characterised by hypoalbuminemia and malnutrition, raising a high suspicion of small bowel pathology.

Clinically, diaphragm disease is often mistaken for Crohn's disease or tuberculosis due to the presence of multiple strictures [4]. The inflammation and ulcers in diaphragm disease are limited to the mucosal layer, unlike Crohn's disease. In our case, the histopathological findings were able to rule out Crohn's disease and tuberculosis in the absence of transmural inflammation and cessating granulomas, respectively. However, other histopathological findings were consistent with diaphragm disease. The active correlation of chronic NSAID use, development of strictures, capsule retention, and collective histopathological findings confirmed diaphragm disease.

This case emphasises the importance of considering small bowel diaphragm disease in patients with prolonged NSAID use and unexplained gastrointestinal symptoms. It highlights the role of surgical resection and definitive management. 

## Conclusions

This unique case of NSAID-induced small bowel diaphragm disease emphasises the importance of recognition of rare complications of NSAID use. This case of gastroduodenal ulceration secondary to NSAIDs highlights the importance of considering diaphragmatic disease when symptoms and signs are suggestive of small bowel malabsorption or subacute obstruction. Early multidisciplinary evaluation and surgical consultation are crucial in diagnosis and management. Surgical treatment remains the definitive therapeutic approach in patients with strictures, especially with small bowel capsule retention. 
